# Karyopherin binding interactions and nuclear import mechanism of nuclear pore complex protein Tpr

**DOI:** 10.1186/1471-2121-10-74

**Published:** 2009-10-16

**Authors:** Iris Ben-Efraim, Phyllis D Frosst, Larry Gerace

**Affiliations:** 1 Department of Cell Biology, The Scripps Research Institute, La Jolla, CA 92037, USA; 2 National Human Genome Research Institute, National Institutes of Health Bethesda, Maryland 20892, USA

## Abstract

**Background:**

Tpr is a large protein with an extended coiled-coil domain that is localized within the nuclear basket of the nuclear pore complex. Previous studies [1] involving antibody microinjection into mammalian cells suggested a role for Tpr in nuclear export of proteins via the CRM1 export receptor. In addition, Tpr was found to co-immunoprecipitate with importins α and β from *Xenopus laevis *egg extracts [2], although the function of this is unresolved. Yeast Mlp1p and Mlp2p, which are homologous to vertebrate Tpr, have been implicated in mRNA surveillance to retain unspliced mRNAs in the nucleus[3,4]. To augment an understanding of the role of Tpr in nucleocytoplasmic trafficking, we explored the interactions of recombinant Tpr with the karyopherins CRM1, importin β and importin α by solid phase binding assays. We also investigated the conditions required for nuclear import of Tpr using an *in vitro *assay.

**Results:**

We found that Tpr binds strongly and specifically to importin α, importin β, and a CRM1 containing trimeric export complex, and that the binding sites for importins α and β are distinct. We also determined that the nuclear import of Tpr is dependent on cytosolic factors and energy and is efficiently mediated by the importin α/β import pathway.

**Conclusion:**

Based on the binding and nuclear import assays, we propose that Tpr is imported into the nucleus by the importin α/β heterodimer. In addition, we suggest that Tpr can serve as a nucleoporin binding site for importin β during import of importin β cargo complexes and/or importin β recycling. Our finding that Tpr bound preferentially to CRM1 in an export complex strengthens the notion that Tpr is involved in protein export.

## Background

Molecules are transported between the cytoplasm and the nucleus through nuclear pore complexes (NPCs), massive proteinaceous structures that span the double membrane of the nuclear envelope (NE). Molecules smaller than ~20-40 kDa in size can passively diffuse through the NPC. However most protein, and nucleic acid is transported by receptor and energy dependent mechanisms (reviewed in [5-8]).

Nucleocytoplasmic transport is mediated by shuttling transport receptors termed karyopherins or importins/exportins (reviewed in [5,7]). In the extensively studied classical nuclear import pathway, cargoes carrying a basic amino acid-rich nuclear localization sequence (NLS) bind to the adaptor importin a, which in turn associates with the import receptor importin β that mediates transport into the nucleus. A second class of import cargo directly binds to importin β in the absence of an adaptor. In the classical nuclear export pathway, cargoes carrying a leucine-rich nuclear export signal (NES) bind to the exportin CRM1 together with RanGTP to be transported out of the nucleus.

The small GTPase Ran, which binds directly to both importins and exportins, plays a key role in determining the directionality of nuclear transport. The GTP-bound form of Ran is concentrated in the nucleus and the GDP-bound form predominates in the cytoplasm, due to the nuclear localization of the Ran guanine nucleotide exchange factor RCC1 (RanGEF) and the cytoplasmic localization of the Ran GTPase- activating protein (RanGAP). The binding of RanGTP to karyopherins modulates the affinity of the receptors for cargo. When an importin-cargo complex encounters RanGTP in the nucleus, RanGTP promotes the dissociation of cargo from the receptor as well as dissociation of the importin from nucleoporins, and the importin-RanGTP complex is recycled back to the cytoplasm. The converse is true for exportins: intranuclear RanGTP promotes the binding of cargo to exportins, and when the RanGTP-containing export complex encounters RanGAP in the cytoplasm, GTP hydrolysis results in release of the cargo and regeneration of the free exportin [9-11].

The framework of the NPC consists of eight central spokes flanked by nuclear and cytoplasmic rings, forming a ring-spoke assembly that surrounds a central transport channel. Extending outward from the ring-spoke assembly are ~50-100-nm-long nuclear fibrils, which are joined in a basket-like structure ("nuclear basket"), and ~35-50-nm-long cytoplasmic fibrils (reviewed in [12,13]). The NPC of both mammals and yeast comprise ~30 different nucleoporins, which are present at integral multiples of 8 copies, consistent with the 8-fold rotational symmetry of the NPC framework. Within the NPC, nucleoporins are typically organized in distinct subcomplexes that are localized to specific regions of the NPC [13]. Approximately 1/3 of the nucleoporins contain multiple copies of the FG (phenylalanine-glycine) di-amino acid repeat. These FG repeats are clustered in domains ("FG domains") that are intrinsically unstructured. The FG domains appear to form the major diffusion barrier of the NPC [14,15], and also serve as the key interaction sites for karyopherins during their transit through the NPC [12,16].

In addition to undergoing reversible disassembly during mitosis in higher eukaryotes, NPCs are assembled throughout interphase in concert with NE growth [17]. Moreover many nucleoporins have intranuclear pools that appear to undergo dynamic exchange with NPC localized forms [18]. It is plausible that many if not most nucleoporins are imported into the nucleus by receptor-mediated pathways, but this process has not been studied in detail.

A conserved component of the NPC is the protein Tpr (for translocated promoter region) [19] and its homologs. Mammalian Tpr is a 267 kDa structurally bipartite protein comprising 2,349 amino acids. Its N-terminal 1,600 residue domain associates in a dimer to form a parallel two-stranded coiled-coil interrupted periodically along its length. The C-terminal domain comprising ~800 amino acids is highly acidic and is predicted to be unstructured [20]. Tpr homologs have been characterized in *Xenopus laevis *[21], *Saccharomyces cerevisiae *(myosin-like proteins 1 and 2; Mlp1p and Mlp2p) [22], *Drosophila melanogaster *[23] and *Arabidopsis thaliana *[24]. In mammalian cells, Tpr is localized to the nucleoplasmic fibrils of the NPC [1,25] and is suggested to act as the main architectural element of the nuclear basket [25]. Mammalian Tpr is tethered to the NPCs through interaction with Nup153 [26], whereas in yeast, Mlp1p and Mlp2p have been suggested to be anchored to the NPC by interactions with Nic96, or with Nup60 [27]. Numerous functions have been attributed to vertebrate Tpr and its yeast homologs Mlp1p and Mlp2p in addition to a role in NPC architecture. These involve mRNA export [27-30], nuclear protein export [1,31], silent telomeric chromatin organization and telomere length control [32-34], spindle pole assembly in yeast [35], unspliced RNA retention [4,36] and localization and stabilization of a desumoylating enzyme Ulp1 [37,38]. In addition, *Drosophila *Tpr has been linked to mitotic spindle organization and spindle checkpoint control [39].

In mammalian cells, classical nuclear protein import is not detectably affected in Tpr depleted cells [1,40], whereas classical nuclear export is found to be significantly inhibited [1]. Yeast cells carrying a double deletion of Mlp1p and Mlp2p display markedly slower import of a model cargo [41], but protein export has not been examined. A biochemical study conducted with *Xenopus Laevis *egg extracts demonstrates that importin β and importin α co-immunoprecipitate with Tpr [2]. Whether this interaction is direct or indirect was not investigated, and its biological significance remains unresolved. To gain further insight into the role of Tpr in nucleocytoplasmic trafficking of protein, we investigated the interaction of Tpr with the karyopherins involved in classical nuclear import and export using quantitative binding assays. Our findings indicate that Tpr binds specifically and with relatively high affinity to these nuclear transport receptors, and support the notion that Tpr provides a docking site for importin α/β and CRM1 in nuclear import and export. Furthermore, the results of our binding analysis together with *in vitro *nuclear import assays indicate that the nuclear import of Tpr is efficiently mediated by the classical importin α/β pathway.

## Results and Discussion

### Characterization of the binding of CRM1, importin β and importin α to Tpr

**Figure 1:**
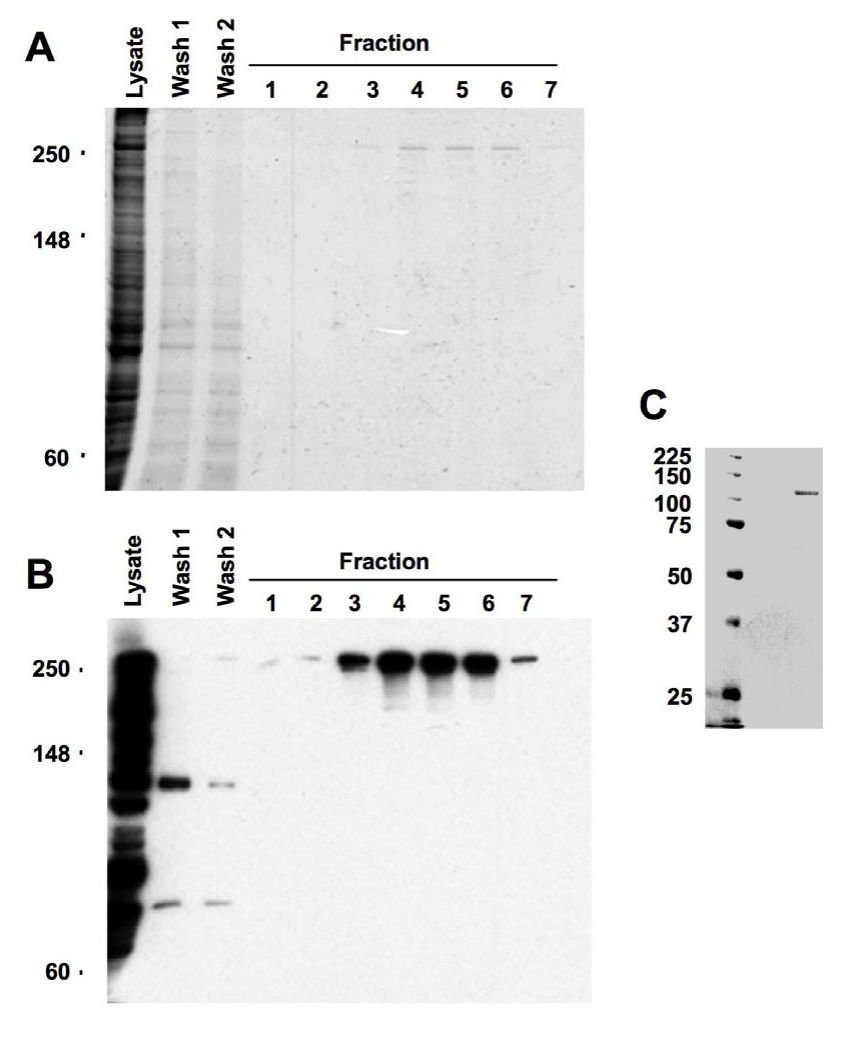
**Purification of recombinant Tpr from SF9 cells and C-Tpr (residues 1626-2348) from bacteria**. Panel A: Coomassie stained SDS-PAGE (8%) of a representative 6×His-Tpr purification. Shown are the cell lysate following centrifugation, washes (800 mM NaCl, 20 mM HEPES pH7.5, 10% glycerol, 15 mM imidazole and protease inhibitors) to remove material non-specifically bound to the resin and seven elution fractions (100 mM NaCl, 20 mM HEPES, 150 mM imidazole and 5 mM beta-mercaptoethanol with protease inhibitors). Panel B: Western analysis of the samples in Fig.1 Panel A, probed with a combination of antibodies to the N-terminus and the C-terminus of Tpr. Panel C: Coomassie stained SDS-PAGE (8%) of a representative purified CTpr(1626-2348).

To investigate the interactions of Tpr with the karyopherins CRM1, importin β and importin α, we used quantitative solid phase binding analysis. To this end, we expressed recombinant full-length mammalian Tpr using a baculovirus system, and expressed the C-terminal domain of Tpr ("C-Tpr", residues 1626-2348) in bacteria. The expression and purification of these Tpr constructs was analyzed by SDS-PAGE (Fig. 1A). Full-length Tpr was found to migrate at its predicted molecular weight of ~250 kDa, and immunoblotting with a mixture of antibodies raised against C and N-terminal segments revealed an intact protein with no apparent proteolytic degradation (Fig. 1B). The migration of purified C-Tpr was in agreement with its calculated molecular weight of 112 kDa (Fig. 1C).

**Figure 2:**
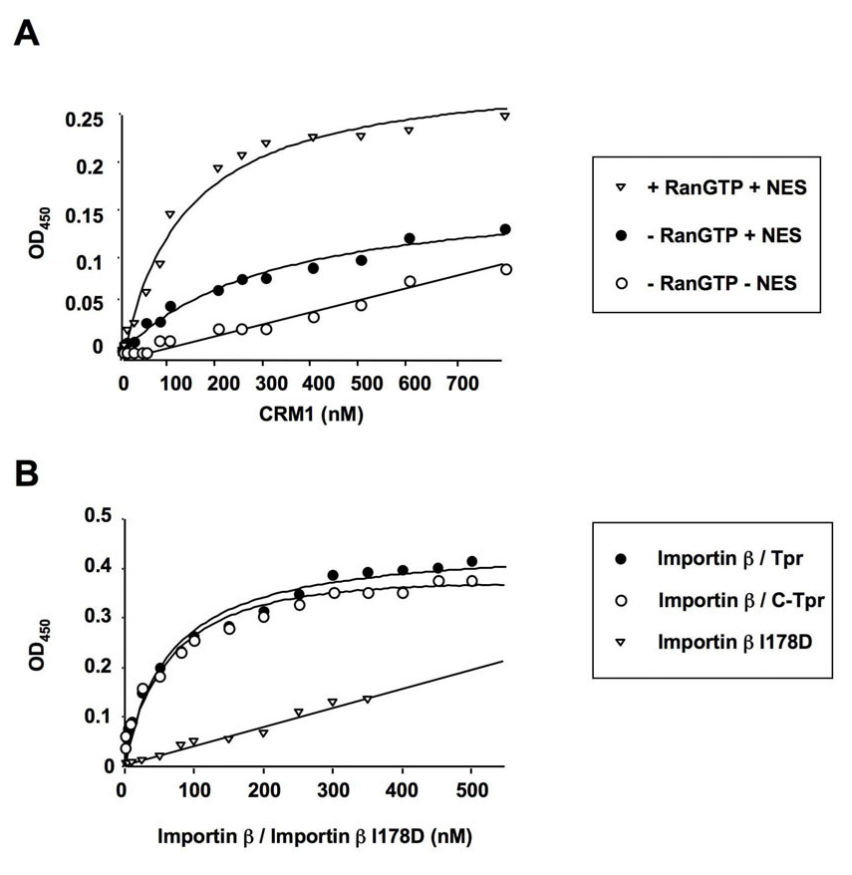
**Characterization of the binding of CRM1 or importin β to Tpr or C-Tpr**. Panel A: Binding was analyzed in the absence of RanGTP and NES (empty circles) or presence of RanGTP and NES (filled triangles) or absence of RanGTP and presence of NES (filled circles). Increasing eqimolar concentrations of CRM1, RanGTP and NES were incubated with Tpr and bound CRM1 was quantified as described in materials and methods. Panel B: Binding to Tpr was analyzed with increasing concentrations of importin β (filled circles) or importin β I178D (inverted triangles). Binding to C-Tpr was analyzed with increasing concentrations of importin β. The results are from duplicates of a single typical experiment. The standard deviation was <5% of the mean. The data were fitted to the equation B(Y) = B_max _× Y/(K_d _+ Y) (using Kaleida Graph software) where Y is the concentration of CRM1 (Panel A) or importin β/importin β I178D (Panel B) and B is the amount of CRM1(Panel A) or importin β/importin β I178D (Panel B) specifically bound. The correlation coefficients of the data to the fitted curves were >0.99.

We first analyzed the interactions of Tpr with CRM1 [1]. Because Tpr is localized to the nucleoplasmic side of the NPC, it could be a binding site for the trimeric CRM1 export complex at an early transport step. Alternatively, Tpr might have an indirect role in export related to receptor recycling or some other function. Since RanGTP and NES have been shown to cooperatively bind to CRM1 and to increase its affinity for nucleoporins that have been linked to export [42-44], we examined whether they would have an effect on the binding of CRM1 to Tpr. Tpr or C-Tpr were adsorbed to microtiter plates and the binding of CRM1 in the presence of NES peptide or NES peptide + RanGTP or absence of both was detected with primay antibodies raised against CRM1 and horseradish peroxidase-conjugated secondary antibodies. We found saturable binding of CRM1 to Tpr in the presence of an NES peptide or an NES peptide + RanGTP (Fig. 2A), although the apparent affinity of CRM1 for Tpr was significantly higher in the presence of NES + RanGTP (K_d _= 83 nM, Table 1) as compared to NES alone (*K*_d _= 176 nM, Table 1). In the absence of NES and RanGTP there was no saturable binding of CRM1 to Tpr in the concentration range analyzed. These results build on our previous finding that CRM1-mediated export is reduced considerably when Tpr is diminished or obstructed [1], and further suggest that Tpr is a docking site for the trimeric CRM1 nuclear export complex. Although Tpr depletion from cells was reported not to affect CRM1-mediated export in other studies [40], this might reflect differences in experimental approaches; e.g., cargo-receptor complex association with Tpr may not have been rate-limiting for export with the conditions used in the latter case [40].

**Table 1:** Dissociation constants for Tpr interaction with: CRM1, importin β or importin α.

**Recombinant Protein**	***K*_d _apparent (nM)**
CRM1	Too low to measure
CRM1+NES	176 ± 8 (3)
CRM1+NES+RanGTP	83 ± 5 (3)
Importin β	63 ± 5 (3)
Importin β I178D	Too low to measure
Importin α	21.5 ± 1.5 (3)

Data represent the mean ± SD (number of repeats in parentheses) for the apparent dissociation constant

*K*_d _(see legend to Figs. 1 and 2).

In studies conducted in our laboratory and others, import of classical NLS cargoes was found to be unaffected in Tpr depleted cells [1,40]. However, a biochemical study conducted with *Xenopus *egg extracts demonstrated that importins α and β co-immunoprecipitate with Tpr [2]. This study did not distinguish whether binding of importin β and importin α to Tpr was direct or via an unidentified bridging factor. Importin β binds directly to several FG repeat nucleoporins with apparent K_d _values between 9 nM and 225 nM depending on the nucleoporin [45], reflecting binding interactions of the importin β import complex with the NPC during translocation. We examined whether Tpr was able to directly bind to importin β and/or importin α in a similar affinity range. The binding between importin β and Tpr (Fig. 2B) showed a saturable binding isotherm with a relatively high apparent affinity (*K*_d _= 50 nM, Table 1), demonstrating a direct interaction between the two proteins. Similar binding was found for C-Tpr (Fig. 2B), indicating that importin β interacts with this region of Tpr.

**Figure 3:**
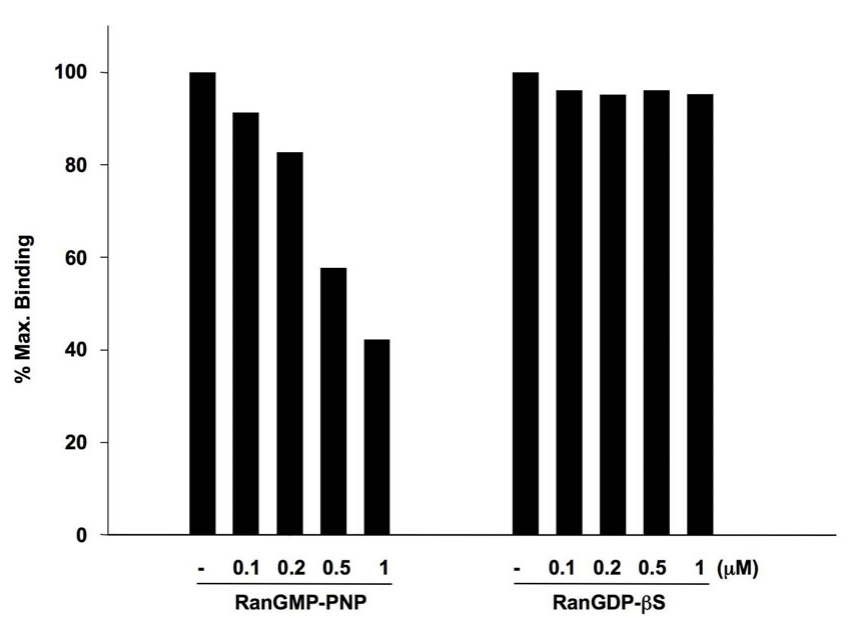
**The binding of importin β to Tpr in the solid phase assay is sensitive to RanGTP**. The binding of 100 nM importin β to Tpr adsorbed to microtiter wells was analyzed in the absence, or in the presence of 0.1, 0.2, 0.5 and 1 μM RanGMP-PNP or RanGDPβS, as indicated. The bound importin β was quantified as described in materials and methods.

Since the interaction of importin β both with cargo and with nucleoporins is dissociated by RanGTP [46], as a specificity control we examined the effect of RanGMP-PNP and RanGDP-βS (Ran bound to nonhydrolyzable analogs of GTP and GDP, respectively) on the binding of importin β to Tpr (Fig. 3). Increasing concentrations of RanGMP-PNP led to progressively less binding of importin β to Tpr, and a 10 fold molar excess reduced binding to 42% of that observed in the absence of RanGMP-PNP (Fig. 3). RanGDP-βS had no significant effect on the interaction of importin β with Tpr. This indicates that RanGTP but not RanGDP decreases the binding of importin β to Tpr, and argues for the biological specificity of the interaction we detected.

Importin β is a superhelical solenoid of tandem HEAT repeats, which binds to nucleoporin FG repeats on its outer surface, and to RanGTP and importin α and other cargoes on its inner surface (reviewed in [7]). Since Tpr is a "cargo" that presumably needs to be imported into nuclei for interphase NPC assembly, the Tpr-importin β interaction that we have detected could reflect a hypothetical cargo-like binding of Tpr to importin β that is importin a-independent. Alternatively, it could reflect a possible FG repeat-like binding of importin β to Tpr involved in importin β trafficking. Indeed, Tpr contains 3 FG repeats in its C-terminal region that could provide such binding sites. To distinguish between these possibilities, we measured the affinity of the mutant importin β I178D for Tpr. This mutant has a decreased affinity for FG repeats of nucleoporins due to disruption of a conserved hydrophobic binding pocket on the outer surface of importin β [16]. However, the mutation has no effect on the binding to either Ran or to various cargoes, including importin α. We could not measure any specific binding of importin β I178D for Tpr in the concentration range tested (Fig. 2B), indicating that the binding of Tpr involves the major FG-repeat binding pocket on the outer surface of importin β. This finding suggests that Tpr functions as a nucleoporin that provides a binding site for importin β during import of importin β-cargo complexes or during recycling of cargo-free importin β, rather than an importin α-independent import cargo.

**Figure 4:**
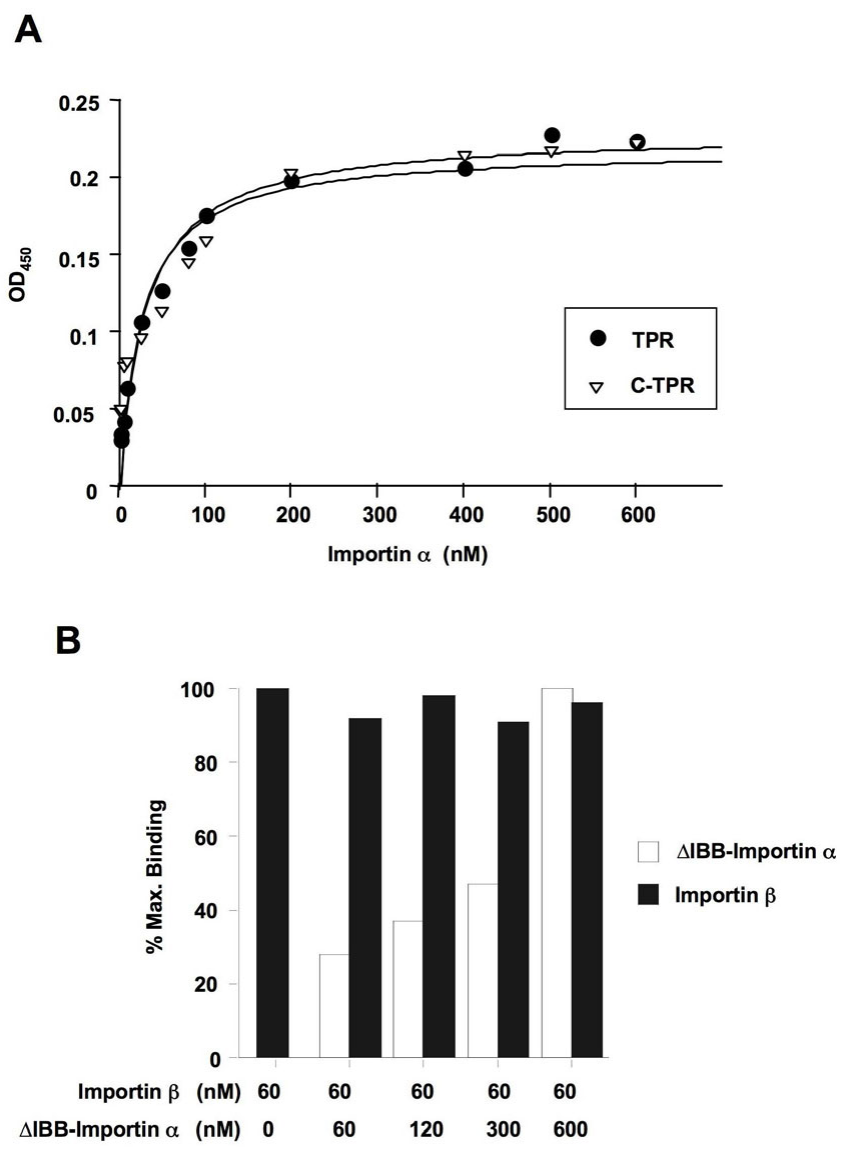
**Characterization of the binding of ΔIBB-importin α to Tpr and demonstration of different binding sites for ΔIBB-importin α and importin β on Tpr**. Panel A: Increasing concentrations of ΔIBB-importin α were incubated with immobilized Tpr or C-Tpr and the bound ΔIBB-importin α was quantified as described in materials and methods. The data were analyzed as described in Fig. 1. Panel B: The binding of 60 nM importin β to Tpr pre-adsorbed to microtiter wells was analyzed in the absence of ΔIBB-importin α or in the presence of 60, 120, 300 and 600 nM ΔIBB-importin α, as indicated. Bound importin β and ΔIBB-importin α were quantified as described in Materials and Methods.

We next measured the affinity of importin α for Tpr and C-Tpr (Fig. 4A). The results showed a saturable binding isotherm with a high apparent affinity (K_d _= 21.5 nM, Table 1) for both full length Tpr and C-Tpr, demonstrating a direct interaction between Tpr and importin α. Similar values were recorded for the interaction between importin α and the SV40 T-antigen [47], a commonly studied cargo for importin α. Since both importin α and importin β are capable of directly binding to Tpr, we investigated whether they can bind to Tpr concurrently or whether they compete for each other's binding. For this analysis, we used an N-terminal deletion mutant of importin α (ΔIBB-importin α) that lacks the importin β binding domain, so that we could examine the binding of importin α to Tpr in the absence of its binding to importin β. We conducted competition-binding assays with importin β kept at a constant concentration of 60 nM (~the K_d _for Tpr) and the ΔIBB-importin α concentration increased from 0-600 nM. As shown in Fig. 4B, the ΔIBB-importin α does not diminish the binding of importin β to Tpr at any concentration. Similar results were obtained with binding experiments that involved either importin α or ΔIBB-importin α held at the constant concentration of 60 nM and importin β varied from 0-600 nM (data not shown). Together, these results indicate that importin a and importin β bind to different sites on Tpr.

### Nuclear Import of C-Tpr is mediated by the importin α/β pathway

**Figure 5:**
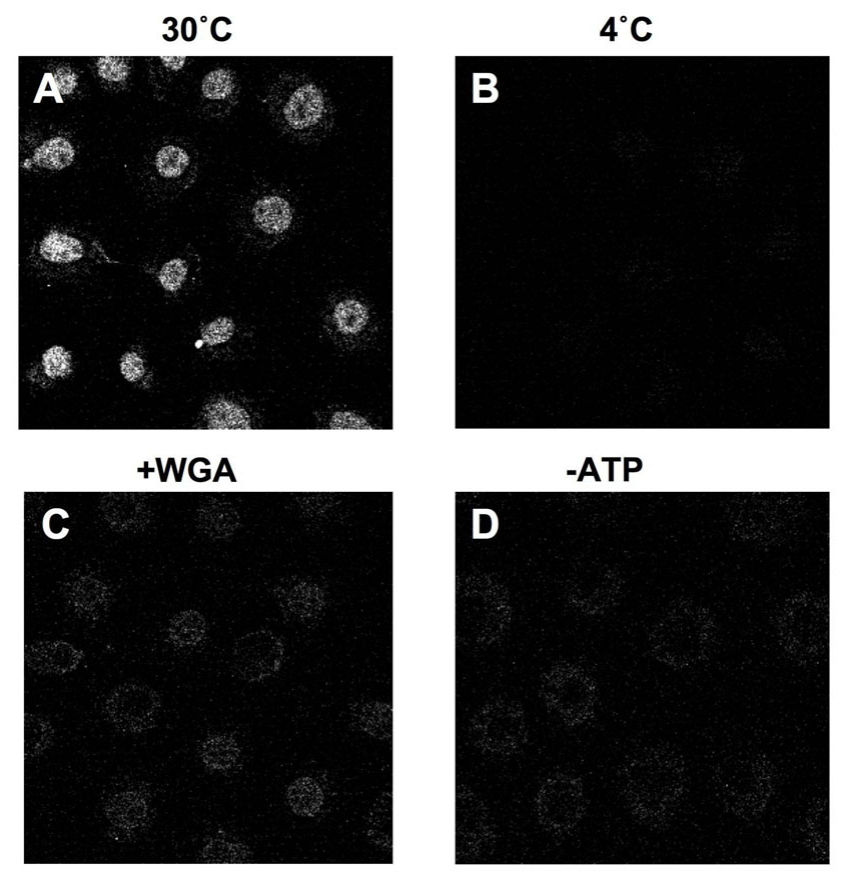
**C-Tpr import into the nucleus is cytosol, temperature and energy dependent and is blocked by WGA**. Digitonin-permeabilized NRK cells were incubated with 8 pmoles of Cy5-C-Tpr for 20 min at 30°C (panel A) or 4°C (panel B) in the presence of cytosol and an energy-regenerating system. Panel C: 8 μg WGA was added. Panel D: the ATP regenerating system was replaced by an ATP-depleting system, 0.8885 U hexokinase + glucose. Samples were visualized by confocal microscopy.

**Figure 6:**
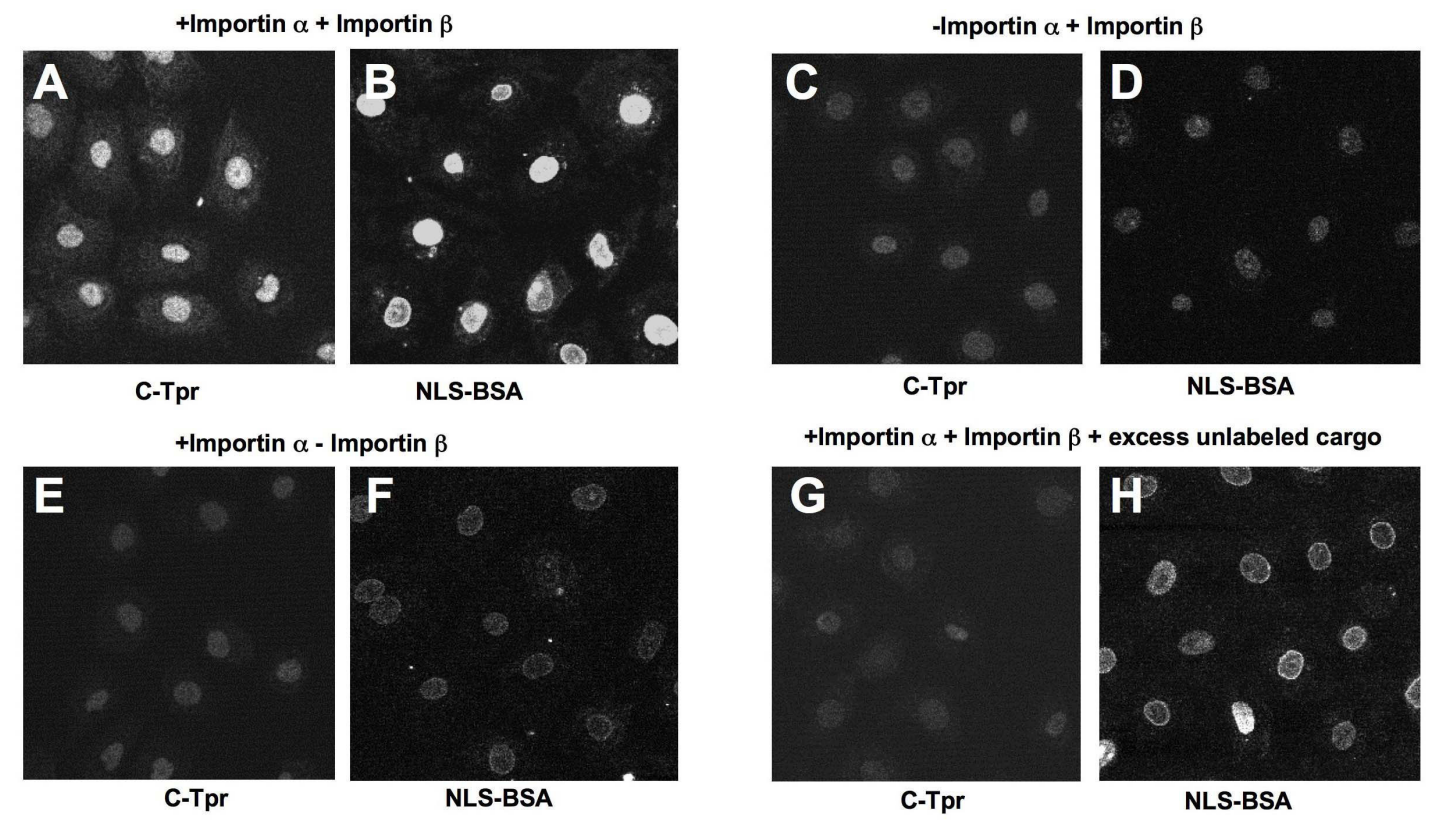
**Role of importin α/β nuclear import pathway in the nuclear distribution of C-Tpr**. Digitonin-permeabilized NRK cells were incubated with 8 pmoles Cy5-C-Tpr (Panels A, C, E, G) or 8 pmoles Cy3-BSA-NLS (coupled with a synthetic peptide comprising the NLS of SV40 T-antigen; Panels B, D, F, H) and nuclear import was conducted with recombinant factors as described in Materials and Methods. Panels A, B: nuclear import of Cy5-C-Tpr and Cy5-BSA-NLS performed at 30°C; panels C, D: importin α was omitted; panels E, F: importin β was omitted; panels G, H: Cy5-C-Tpr and Cy3-BSA-NLS competed with 10-fold molar excess of unlabeled cargo. Samples were visualized by confocal fluorescent microscopy.

Previous studies showed that the C-terminal domain of Tpr is responsible for its nuclear localization [41,28,48]. A minimal segment conferring nuclear localization is found in residues 1812-1867, and this contains a sequence resembling the bipartite basic amino acid NLS that is recognized by importin α [48]. These results, combined with our data showing that C-Tpr binds to importin α with a cargo-like affinity, suggest that the binding of Tpr to importin α could be important for Tpr import into the nucleus. We tested this hypothesis using digitonin-permeabilized NRK cells supplemented with exogenous cytosol to examine the nuclear import of the C-Tpr fragment. Nuclear import was visualized by the concentration of fluorescent cargo in the nucleus. NRK cells grow as flat, extensively spread cells that are 2-3 times the diameter of the nucleus (e.g. see Fig. 5, where fluorescent cargo is adsorbed to cytoplasmic structures). Full-length recombinant Tpr was unsuitable for this work, since it extensively aggregated during labeling and in the permeabilized cell assay (data not shown). Substantial nuclear accumulation of C-Tpr was detected when permeabilized cells were supplemented with cytosol and an energy-regenerating system at 30°C (Fig. 6). Nuclear import of C-Tpr was inhibited when the reaction was carried out at 4°C (Fig. 6) or when it was energy-depleted by treatment with hexokinase + glucose (Fig. 6). WGA, a lectin that binds to the NPC and that inhibits receptor-mediated transport, also inhibited the nuclear import of C-Tpr (Fig. 6). Collectively, these results indicate that nuclear accumulation of C-Tpr involves a temperature, energy and NPC-dependent process.

The potential involvement of importin α and importin β in the nuclear import of C-Tpr was studied in digitonin-permeabilized cells reconstituted with recombinant transport factors (Fig. 5). We used the import of BSA-NLS of SV40 T-antigen, which is imported by the importin α/β pathway, as a positive control. Nuclear import of C-Tpr was observed when the permeabilized cells were supplemented with exogenous recombinant importin α/β (Fig. 5) and was considerably diminished when either importin α or importin β were omitted (Fig. 5), similar to the control cargo BSA-NLS, which requires both importins α and β for nuclear import (Fig. 5). Nuclear import of fluorescently labeled C-Tpr was strongly diminished with a 10-fold molar excess of unlabeled C-Tpr (Fig. 5). Together these data support our main conclusion that a combination of importin α and importin β are sufficient for efficient nuclear import of C-Tpr. This import pathway could be involved in Tpr assembly in the NPC during interphase, as well as at the end of mitosis. Since assembly of Tpr in the NPC at the end of mitosis follows that of almost all other nucleoporins [49], it is likely that Tpr enters the nucleus through largely if not completely intact NPC and thus it plausibly utilizes a receptor-mediated pathway to enter the nucleus.

## Conclusion

Our quantitative binding studies showing that Tpr selectively binds to CRM1 present in a trimeric export complex supports the notion that Tpr plays a direct role in protein export [1]. Our binding studies further suggest that Tpr can provide a binding site for the import and/or recycling of importin β complexes. Finally, the results presented here establish that nuclear import of Tpr can efficiently occur by the importin α/β import pathway.

## Methods

### Plasmid Construction

Recombinant baculovirus particles containing the mammalian Tpr cDNA were prepared using the Bac-to-Bac Baculovirus Expression System (Gibco BRL). The cDNA of Tpr was cloned into the Sal I site of the donor plasmid pFastBac HTc containing an N-terminal 6×His tag. The recombinant donor plasmid was transformed into DH10Bac *Escherichia coli *containing the viral backbone, where recombination generated a recombinant bacmid containing His-Tpr inserted into the viral backbone. Mammalian C-Tpr encompassing amino acids 1626-2348 was constructed by inserting HindIII fragment into HindIII site of pET30c vector, and the construct was analyzed for correct orientation.

### Expression and Purification of Recombinant Proteins

N-terminal His-tagged Tpr was expressed in SF9 cells at 27°C for 45 hours. Cells were lysed in RIPA buffer (1% NP-40, 1% sodium deoxycholate, 0.1% SDS, 150 mM NaCl, 10 mM sodium phosphate, pH 7.2, 2 mM beta-mercaptoethanol and 2 mM EDTA) with mammalian protease inhibitor cocktail (1:100, Sigma). Cell lysates were cleared at 14,000 × g and supernatants were purified on Talon affinity resin (Clontech). Only micorgram quantities of full-length Tpr could be obtained by this method. From Western blot analysis, we found that a significant amount of an immunoreactive band comigrating with purified full-length Tpr did not bind to the Talon resin, suggesting either that the His-tag was cleaved from this fraction, or that the His-tag was inaccessible, possibly because Tpr in the unbound fraction was present in small aggregates. Recombinant C-Tpr (comprising residues 1626-2348) was expressed in *Escherichia coli *BL21^+ ^cells and grown at 37°C in 2 × LB medium to an OD_600 _of 0.5-0.6. Expression was induced by the addition of 0.2 mM IPTG for 3 h at 37°C. Cells were resuspended in 50 mM Tris buffer, pH 7.5 supplemented with 300 mM NaCl, 2 mM DTT, 1 mg/ml lysozyme, 10 μg/ml DNase, and the protease inhibitor cocktail. The suspension was sonicated (3 × 20 s), and centrifuged at 100,000 × *g *for 30 min. The supernatant was loaded on a Sepharose Q column, washed extensively with the lysis buffer containing 300 mM NaCl, and then eluted with a gradient of 300-530 mM NaCl for 1 hour. Fractions containing C-Tpr were pooled; ammonium sulfate precipitated and run on a Superdex 75 column equilibrated in transport buffer. Expression and purification of His-importin α, ΔIBB-importin a, importin β, His-S-tag-importin β, Ran, nuclear transport factor 2 (NTF2) and GST-M9 (a cargo of the nuclear import receptor, transportin) was performed as previously described [50]. These proteins were dialyzed into transport buffer [51] before use in the various experiments.

### Microtiter Plate Binding Assay

Solid phase binding assays were carried out on microtiter plates (Maxisorp; Nunc) coated with 25 ng of Tpr or C-Tpr. Assays were conducted as previously described [45]. Binding of His-importin α to Tpr or C-Tpr immobilized on microtiter plates was detected by an affinity-purified rabbit pAb raised against human His-importin α. Binding of His-S-tag-importin β to Tpr or to C-Tpr on microtiter plates was detected by an affinity-purified pAb raised against S-tag. Binding of CRM1 to Tpr in the presence or absence of a synthetic peptide comprising the NES derived from PKI (ELALKLAGLDIN) [52] and RanGTP was detected by affinity purified rabbit pAb raised against a C-terminal peptide derived from CRM1. Horseradish peroxidase-conjugated secondary antibodies were used for colorimetric detection (Pierce Chemical Co) using 3,3', 5,5'-tetramethylbenzidine as substrate (Calbiochem). The three components: CRM1, NES and RanGTP were used at equimolar concentrations in the binding experiments.

### Competition-binding assay

Competition assays between ΔIBB-importin α and importin β for binding to Tpr were carried out on microtiter plates (Maxisorp; Nunc) coated with 25 ng Tpr. Different ratios of ΔIBB-importin a and importin β were mixed together and incubated with immobilized Tpr. The experiments were carried out in duplicates. One repeat was probed with antibodies against S-tag to detect importin β and the other repeat was probed with antibodies against ΔIBB-importin α. Results are presented as percentage of highest level of binding.

### Nuclear Import Assay

For analysis of nuclear import in digitonin-permeabilized adherent NRK cells, nuclear import *in vitro *assays, substrate visualization, and preparation of HeLa cytosol (by digitonin lysis) were carried out essentially as in [50]. 5 mg of C-Tpr was coupled with Cy5™ (1 vial, Amersham Pharmacia Biotech. Inc.) for 30 minutes at room temperature and then separated from free dye by chromatography on a PD10 column (Amersham Pharmacia Biotech. Inc.) pre-equilibrated with transport buffer. Labeling of BSA with Cy3™ was performed similarly. Conjugation of labeled BSA to a peptide derived from the NLS of SV40 T- antigen (CGGGPKKKRKVEDI) was performed as in [51]. Import reactions contained either 2.5 mg/ml HeLa cytosol or recombinant factors at the following concentrations: 100 nM His-importin α, 62.5 nM importin β, 450 n Ran, 500 nM NTF2. The import reaction mixture was supplemented with an energy-regenerating system and 1 mM GTP.

## List of Abbreviations

BSA: bovine serum albumin; CRM1: chromosome region maintenance 1; FG: phenylalanine glycine; Mlp1p: myosin-like protein 1; NE: nuclear envelope; NES: nuclear export signal; NLS: nuclear localization sequence; NPC: nuclear pore complex; NTF2: nuclear transport factor 2; pAb: polyclonal antibody; RanBP1: RanGTP binding protein; RanGAP1: RanGTPase activating protein; RanGEF: Ran guanine nucleotide exchange factor; RCC1, regulator of chromosome condensation 1; Tpr: translocated promoter region; WGA: wheat germ agglutinin.

## Authors' contributions

IBE designed and performed the experiments, analyzed and interpreted data, and drafted the figures and manuscript. PDF prepared recombinant full-lengthTpr, performed preliminary versions of the binding assays and contributed intellectually to this development of the experiments. LG conceived and oversaw the conduct of the research and was involved in writing the manuscript. All authors read and approved the final manuscript.
